# Patient‐size based dose optimization in projection radiography examinations: A BMI‐guided approach

**DOI:** 10.1002/acm2.70191

**Published:** 2025-07-15

**Authors:** Sachith Welarathna, Sivakumar Velautham, Sivananthan Sarasanandarajah

**Affiliations:** ^1^ Department of Physics University of Peradeniya Peradeniya Sri Lanka; ^2^ Postgraduate Institute of Science University of Peradeniya Peradeniya Sri Lanka; ^3^ School of Health and Biomedical Sciences RMIT University Melbourne VIC Australia; ^4^ Department of Physical Sciences Peter MacCallum Cancer Centre Melbourne VIC Australia; ^5^ Department of Medical Physics Bharathiar University Coimbatore India

**Keywords:** benchmark doses (BMDs), BMI, dose optimization, kerma‐area product (P_KA_), patient protection, projection radiography

## Abstract

**Purpose:**

The increasing prevalence of obesity poses challenges for dose optimization in projection radiography due to greater anatomical thickness in overweight and obese patients worldwide. Diagnostic reference levels (DRLs) alone may not adequately account for variations in body habitus, potentially leading to suboptimal patient protection. This study aimed to explore benchmark doses (BMDs) based on patient body mass index (BMI) for projection radiography examinations of major anatomical regions in Sri Lanka, providing a complementary approach for dose optimization alongside DRLs.

**Methods:**

This prospective study included 1989 adult patients (≥18 years) undergoing abdomen anteroposterior (AP), chest posteroanterior (PA), kidney–ureter–bladder (KUB) AP, lumbar spine AP, lumbar spine lateral (LAT), and pelvis AP examinations at six major tertiary care hospitals. For each examination, patient demographics (age, weight, height, and BMI) and exposure parameters (kilovoltage peak [kVp] and tube current‐exposure time product [mAs]) were recorded, and the patient doses in terms of kerma‐area product (P_KA_) were measured using a P_KA_ meter. DRLs (achievable doses) were proposed at the median of the median P_KA_ distribution across hospitals for a standard‐sized patient group (58 ± 20 kg). For BMI‐based BMDs, patients were classified into four standard BMI categories: underweight, normal weight, overweight, and obese. The median P_KA_ distributions across hospitals were used to formulate BMI‐based BMDs, which were then compared with the proposed DRLs for the standard‐sized patient group.

**Results:**

The results showed a progressive increase in BMI‐based BMDs across BMI categories for all examinations studied. BMI‐based BMDs (in Gy.cm^2^) for underweight, normal weight, overweight, and obese patients were as follows: 1.46, 1.94, 2.88, 3.00 (abdomen AP); 0.17, 0.21, 0.22, 0.25 (chest PA); 1.70, 1.76, 2.30, 3.60 (KUB AP); 1.00, 1.03, 1.29, 1.48 (lumbar spine AP); 1.94, 2.09, 2.57, 2.56 (lumbar spine LAT); and 0.60, 1.85, 1.86, 2.24 (pelvis AP). Compared to normal weight patients, underweight patients exhibited percentage reductions in BMI‐based BMDs of 24.7%, 3.4%, 2.9%, 7.1%, 4.5%, and 67.6% for abdomen AP, KUB AP, lumbar spine AP, lumbar spine LAT, chest PA, and pelvis AP, respectively. Conversely, overweight patients demonstrated percentage increases of 48.5%, 30.7%, 25.2%, 23.0%, 4.8%, and 0.5% across the same examinations, while obese patients showed increases of 54.6%, 104.5%, 51.5%, 22.5%, 19.0%, and 21.1%, respectively. DRLs for the standard‐sized patient group were 1.82, 0.22, 2.03, 1.27, 2.21, and 1.90 Gy.cm^2^, respectively.

**Conclusion:**

These findings underscore the importance of BMI‐based BMDs as an effective tool for personalized dose optimization, accounting for variations in patient body habitus. Their integration into clinical practice, alongside DRLs, could enhance patient protection and promote good radiographic practices. Furthermore, the findings underscore the need for the introduction of international guidelines for DRLs in intervals of BMI to ensure standardized implementation across countries.

## 1 INTRODUCTION

Projection radiography has significantly advanced global healthcare, establishing itself as a cornerstone of medical imaging modalities.^1^ Over the past two decades, international radiation protection organizations, such as the International Commission on Radiological Protection (ICRP) and the International Atomic Energy Agency (IAEA), have made substantial progress in optimizing patient doses and improving patient protection in medical radiological examinations.^2^, ^3^, ^4^, ^5^ These efforts, combined with technological advancements, have led to a reduction in the global average annual effective dose per person, from 0.65 mSv in 2006 to 0.56 mSv in 2016.^6^, ^7^ Despite these advancements, the unjustified and suboptimal use of radiological examinations continues to pose stochastic risks, necessitating careful justification to ensure clinical benefits outweigh potential harms.^8^, ^9^ Consequently, routine monitoring and optimization of patient doses remain essential for continuous improvements in patient protection.^2^, ^4^ Medical radiological examinations should be performed to obtain clinically relevant information that supports patient care decisions. However, failure to meet this objective can undermine diagnostic confidence and increase the risk of misdiagnosis.^4^ To mitigate these challenges, it is essential to implement patient‐specific optimization in routine radiographic practice, and reference dose levels should also be tailored accordingly to maximize clinical effectiveness.^5^

The global prevalence of both underweight and obesity in adults has risen steadily from 1990 to 2022, with obesity now surpassing underweight as a predominant public health concern.^10^ In accordance with the World Health Organization (WHO), 2.5 billion adults aged 18 years and above were classified as overweight in 2022, representing 43% of the global adult population, with over 890 million adults identified as obese.^11^ In Sri Lanka, 27.7% of adults are categorized as overweight and 9.6% as obese.^12^ These trends present unique challenges in dose optimization, particularly for overweight and obese patients, due to the increased anatomical thickness to be imaged compared to those of underweight and normal weight patients.^13^, ^14^, ^15^ Therefore, imaging techniques should be optimized to accommodate variations in patient habitus, including accurate recognition of overweight and obese patients to select optimal exposure factors, ensure good radiographic practice, and maintain doses at “as low as reasonably practicable” (ALARP) levels.^14^, ^16^, ^17^ Studies have emphasized the increased radiation‐induced lifetime cancer risks associated with patients with higher body mass index (BMI).^18^ For instance, Alqahtani et al. (2019) reported that the lifetime cancer risk due to radiation exposure could be up to 153% higher in obese patients compared to those with normal weight.^18^ These findings underscore the importance of BMI‐specific dose optimization, which has gained attention in recent years.^13^, ^19^

In general, a higher BMI results in greater photon attenuation, requiring higher exposure parameters, which in turn leads to increased radiation doses, reduced contrast resolution, and higher image noise.^15^, ^20^ Conversely, radiographers using manual exposure control (MEC) mode face challenges when imaging underweight, overweight, or obese patients, as they must carefully select exposure parameters tailored to individual body habitus to ensure optimal protection. However, this requirement further increases the complexity of dose optimization. The ICRP has introduced diagnostic reference levels (DRLs) as effective tools for dose optimization in radiological examinations.^2^ However, DRLs are primarily defined for standard‐sized patients with predefined weight criteria (70 ± 10 kg or 70 ± 20 kg).^2^ Even though standard weight‐based DRLs play a pivotal role in dose optimization, they do not adequately account for variations in body habitus, particularly in overweight and obese patients.^13^, ^20^ In clinical practice, a substantial proportion of patients (48.7% in this study) undergoing projection radiography examinations fall into the underweight, overweight, or obese categories. These patients may not fit within predefined weight‐based DRL categories, leading to the possibility of receiving doses that result in either underexposure or overexposure.^17^, ^21^ BMI‐based benchmark doses (BMDs) offer a practical solution to this issue by enabling dose optimization tailored to variations in patient body habitus and composition; however, image quality should also be considered.^17^, ^19^, ^21^, ^22^

Studies have demonstrated that acceptable quality dose (AQD) provides a better representation of anatomical variability and dose escalation trends compared to DRLs.^21^ However, using BMI‐based BMDs allows for more accurate dose comparisons and adjustments tailored to different body types, which is particularly beneficial for underweight, overweight, and obese patients.^19^ Therefore, integrating BMI‐based BMDs alongside DRLs enables radiographers to balance the diagnostic quality of radiographs with radiation protection across all patient groups.^17^ Despite the growing attention to dose optimization, a few studies have proposed DRLs for Sri Lanka, but these have yet to be established by regulatory bodies.^23^, ^24^, ^25^ In addition, BMI‐based BMDs have not been studied, presenting challenges for dose optimization, particularly in overweight and obese patients. Few studies have investigated the impact of BMI on the kerma‐area product (P_KA_) in projection radiography worldwide, and no BMI‐based DRLs have been established, highlighting a significant knowledge gap.^13^, ^20^, ^26^, ^27^ Vieira et al. (2022) have reported only local DRLs based on patient BMI for selected interventional procedures.^19^ Given Sri Lanka's unique patient demographics and radiographic practices, the formulation of BMI‐based BMDs could significantly improve radiographic practices across BMI categories. This first large‐scale study aims to explore BMI‐based BMDs for projection radiography examinations in major anatomical regions and compare them with proposed DRLs (achievable doses) in Sri Lanka. BMI‐based BMDs can provide a safer, more personalized approach for different BMI groups, allowing radiology professionals to tailor patient doses based on body habitus rather than applying a one size fits all patients standard to a patient group with predefined weight criteria.^17^ The findings from this study are expected to serve as a foundation for developing DRLs in intervals of BMI and strengthening radiation protection practices not only in Sri Lanka but also in other countries seeking to implement similar standards.

## 2 METHODS

### 2.1 Study design and settings

This prospective study was conducted between January 2022 and June 2023 in the radiology departments of six major public tertiary care hospitals in Sri Lanka, representing five of the nine provinces. These hospitals were selected based on their annual imaging workload and the population density of their respective provinces. Data were collected using a single x‐ray system in each hospital.

### 2.2 Study sample, inclusion, and exclusion criteria

Data were collected from 1989 adult patients (aged ≥18 years) who underwent projection radiography examinations of the abdomen, chest, kidney–ureter–bladder (KUB), lumbar spine, and pelvis. Following the International Code of Practice for Dosimetry in Diagnostic Radiology, patient demographic information (age, sex, weight, and height) was recorded, and BMI was calculated.^28^ Patients were classified into BMI categories based on WHO and the Centers for Disease Control and Prevention (CDC, USA) guidelines: underweight (<18.5 kg m^−2^), normal weight (18.5–24.99 kg m^−2^), overweight (25–29.99 kg m^−2^), and obese (>30 kg m^−2^).^11^, ^29^ Patients with excessive body weights (>100 kg), critically ill patients, and pregnant women were excluded from the analysis.

### 2.3 Types of x‐ray examinations

This study examined the following projection radiography examinations: abdomen anteroposterior (AP), chest posteroanterior (PA), KUB AP, lumbar spine AP, lumbar spine lateral (LAT), and pelvis AP. The abdomen AP, KUB AP, and pelvis AP examinations were performed in the supine position, while lumbar spine AP and LAT were conducted in the supine and lateral positions, respectively, in relation to the x‐ray table. Chest PA examinations were performed with patients in the standing position. Only examinations with diagnostically acceptable radiographs, as determined by the radiographers and radiologists in the respective hospitals, were included. Moreover, no repeated or rejected examinations were included. The focus‐to‐detector distance was set at 100 cm for all examinations, except for the chest PA, which was set at 180 cm.

### 2.4 X‐ray systems and exposure parameters

Six x‐ray systems, manufactured by the Shimadzu Corporation (Japan), were used. These included computed radiography (CR) and digital radiography (DR) systems. DR systems featured flat‐panel detectors for direct digital image acquisition, while CR systems used phosphor imaging plates (IP cassette type: CC) scanned by separate reading devices (model: FCR CAPSULA XLII image reader unit manufactured by Fujifilm). All hospitals used the same x‐ray generator model (UD150L‐40E). Machine‐specific information, including type, model, total filtration, and year of manufacture, was recorded (Table 1). Exposure parameters, including kilovoltage peak (kVp) and the product of tube current and exposure time (mAs), were documented for each examination. All systems operated in MEC mode with fixed filtration and anti‐scatter grid configurations. Therefore, the exposure parameters (kVp and mAs) for all examinations were selected by radiographers in each hospital according to the local protocol. These systems were certified for clinical use by the Sri Lanka Atomic Energy Regulatory Council (SLAERC).

**TABLE 1:** Technical specifications of x‐ray systems across hospitals.

Model	Type	Year of manufacture	Total filtration (mm Al equivalent)	Maximum voltage output
RADspeed	CR	2008	3.0	150 kV, 200 mA
RADspeed Pro	CR	2017	2.5	150 kV, 200 mA
RADspeed Pro	CR	2017	2.0	150 kV, 200 mA
RADspeed	DR	2011	2.5	150 kV, 200 mA
RADspeed Pro	DR	2018	2.5	150 kV, 200 mA
RADspeed	DR	2011	2.0	150 kV, 200 mA

Abbreviations: CR, computed radiography; DR, digital radiography.

### 2.5 Dosimetry measurements

DRLs were expressed in terms of P_KA_ in Gy.cm^2^, considering the ICRP guidelines.^2^ P_KA_ values were measured using a VacuDAP Bluetooth P_KA_ meter (VacuTec Meßtechnik GmbH, Germany). This device features a plane transmission ionization chamber with a resolution of 0.01 µGy m^2^ and an active area of 147 × 147 mm^2^. During measurements, the P_KA_ meter was positioned perpendicular to the beam axis beneath the x‐ray collimator exit window. The specified correction factor of 1 was applied based on the manufacturer's response correction graph for the measured energy range of 58–120 kVp.

### 2.6 Ethical approval

Ethical approval was obtained from the Ethics Review Committee of the National Hospital of Sri Lanka (AAJ/ETH/COM/2022), and administrative approvals were obtained from all participating hospitals prior to data collection. Patients were informed about the study objectives, and verbal informed consent was obtained before participation. No patients declined to participate.

### 2.7 Statistical analysis

Descriptive statistics were computed for exposure parameters and P_KA_ values across BMI groups. The normality of distributions for kVp, mAs, and P_KA_ was assessed using the Shapiro–Wilk test. Differences in P_KA_, kVp, and mAs across BMI groups were analyzed using a one‐way analysis of variance (ANOVA) for normally distributed data and the Kruskal–Wallis test for non‐normally distributed data. BMI‐based BMDs were formulated as the median (50th percentile) P_KA_ values for underweight (<18.5 kg m^−2^), normal weight (18.5–24.99 kg m^−2^), overweight (25–29.99 kg m^−2^), and obese (>30 kg m^−2^) groups. BMI‐based BMDs were compared with DRLs (achievable doses), which were proposed at the median of the median dose distribution among hospitals. Statistical analyses were performed using Python (version 3.11.11) in Jupyter Notebook (version 7.0.8), using libraries such as Pandas (version 2.2.2), NumPy (version 1.26.4), Matplotlib (version 3.10.0), Seaborn (version 0.13.2), and SciPy (version 1.13.1), with a significance level of *p* < 0.05 applied for all tests.

### 2.8 Comparison with standard weight‐based DRLs

The formulated BMI‐based BMDs were compared with DRLs based on the weight criterion of 58 ± 20 kg.^23^, ^24^ The DRLs were determined as follows: institutional DRLs (IDRLs) or typical values as the 50th percentile of the P_KA_ distribution for each hospital, and DRLs (achievable doses) as the median (50th percentile) of the median P_KA_ values across hospitals.^23^, ^24^, ^25^

## 3 RESULTS

The study examined a total of 1989 adult patients, categorized into different BMI groups, including underweight, normal weight, overweight, and obese, to formulate BMI‐based BMDs. The mean ± standard deviation (SD) and ranges for the study patient group were as follows: age, 53.5 years (18–94); weight, 57.5 kg (26–100); and BMI, 23.2 kg m^−2^ (12.6–46.3).

Table 2 summarizes the descriptive statistics for kVp, mAs, and P_KA_ across BMI categories for projection radiography examinations of abdomen AP, KUB AP, lumbar spine AP, lumbar spine LAT, chest PA, and pelvis AP. The Shapiro–Wilk test indicated that the distributions of kVp, mAs, and P_KA_ deviated from normality, except for mAs in lumbar spine LAT and pelvis AP examinations. Statistical analysis using the Kruskal–Wallis and one‐way ANOVA tests demonstrated significant differences (*p* < 0.0001) in mean P_KA_ and mAs across BMI categories for abdomen AP, chest PA, KUB AP, lumbar spine AP, lumbar spine LAT, and pelvis AP examinations. Furthermore, significant differences (*p* < 0.0001) in mean kVp were also identified among BMI groups for all examinations, except for abdomen AP (*p* = 0.1872).

**TABLE 2:** Descriptive statistics for kVp, mAs, and P_KA_ across underweight, normal weight, overweight, and obese patient groups (*N* = 1,989).

			P_KA_ (Gy.cm^2^)				kVp				mAs			
Examination	BMI (kg m^−2^)	*N*	Mean	SD	BMDs	*p*‐value	Mean	SD	Median	*p*‐value	Mean	SD	Median	*p*‐value
Abdomen AP (*N* = 86)	Underweight	17	1.56	0.60	1.46	*p* < 0.0000^b^	70.4	4.4	70	*p* = 0.1872^b^	30.4	7.2	28.0	*p* < 0.0000^b^
	Normal Weight	49	2.06	0.73	1.94		70.5	4.7	70		39.1	11.5	36.0	
	Overweight	15	2.84	0.66	2.88		71.7	3.7	72		45.3	9.0	45.0	
	Obese	5	3.07	0.58	3.00		73.8	2.8	75		48.2	6.0	50.0	
KUB AP (*N* = 198)	Underweight	15	1.54	0.52	1.70	*p* < 0.0000^b^	69.7	3.9	70	*p* = 0.0049^b^	33.1	6.7	32.0	*p* < 0.0000^b^
	Normal Weight	110	1.83	0.60	1.76		69.8	5.1	70		36.8	7.9	36.0	
	Overweight	57	2.33	0.75	2.30		71.4	4.0	70		40.9	9.3	40.0	
	Obese	16	3.38	0.81	3.60		74.8	5.0	75		51.5	8.2	53.0	
LS AP (*N* = 287)	Underweight	35	1.01	0.36	1.00	*p* < 0.0000^b^	70.2	5.8	70	*p* < 0.0001^b^	34.1	9.3	36.0	*p* = 0.0002^b^
	Normal Weight	145	1.13	0.45	1.03		71.0	4.1	70		36.4	9.7	36.0	
	Overweight	81	1.43	0.57	1.29		72.6	4.2	73		40.5	10.4	40.0	
	Obese	26	1.60	0.58	1.48		73.9	5.0	75		43.0	10.1	45.0	
LS LAT (*N* = 300)	Underweight	38	2.16	0.95	1.94	*p* = 0.0004^b^	76.1	3.8	75	*p* = 0.0023^b^	57.7	19.8	59.5	*p* = 0.0302^a^
	Normal Weight	147	2.40	1.02	2.09		75.4	4.4	75		62.4	21.8	63.0	
	Overweight	87	2.86	1.16	2.57		77.0	4.1	78		69.2	24.9	63.0	
	Obese	28	2.99	1.35	2.56		77.9	4.7	78		68.2	23.1	71.0	
Chest PA (*N* = 1060)	Underweight	195	0.18	0.09	0.17	*p* < 0.0000^b^	100.3	7.7	100	*p* < 0.0000^b^	4.6	1.8	5.0	*p* < 0.0000^b^
	Normal Weight	536	0.23	0.11	0.21		101.1	6.8	102		5.0	1.6	5.0	
	Overweight	265	0.27	0.16	0.22		102.7	5.8	104		5.4	2.4	5.0	
	Obese	64	0.26	0.12	0.25		104.5	5.6	105		5.2	1.8	5.0	
Pelvis AP (*N* = 58)	Underweight	7	0.91	0.44	0.60	*p* = 0.0038^b^	64.0	3.2	62	*p* = 0.0036^b^	23.6	6.6	20.0	*p* = 0.0053^a^
	Normal Weight	33	1.87	0.77	1.85		70.0	4.3	70		32.7	8.4	32.0	
	Overweight	14	2.21	0.82	1.86		71.7	2.9	72		39.3	12.4	36.0	
	Obese	4	2.45	1.35	2.24		72.3	10.3	72		41.8	18.3	41.0	

^a^ One‐way ANOVA;

^b^ Kruskal–Wallis

Abbreviations: AP, anteroposterior; BMD, benchmark dose; BMI, body mass index; KUB, kidney–ureter–bladder; kVp, kilovoltage peak; LAT, lateral; LS, lumbar spine; mAs, product of tube current and exposure time; PA, posteroanterior; P_KA_, kerma‐area product; SD, standard deviation.

The mean P_KA_ and kVp demonstrated a progressive increase across BMI groups, ranging from underweight to obese categories. Similarly, mAs values exhibited an upward trend across most BMI groups. However, slight decreases were noted in lumbar spine LAT (overweight: 69.2, obese: 68.2) and chest PA (overweight: 5.4, obese: 5.2) due to differences in local practices followed in hospitals.

## 4 DISCUSSION

### 4.1 Influence of BMI on exposure parameters and patient doses

This study highlights the influence of BMI on radiographic exposure parameters and patient doses in projection radiography examinations across major anatomical regions. It is well‐documented that patient doses during these examinations vary substantially among patients with different body habitus due to variations in shape and composition.^20^, ^26^ Therefore, understanding these relationships is important for effective patient dose optimization.

**FIGURE 1:**
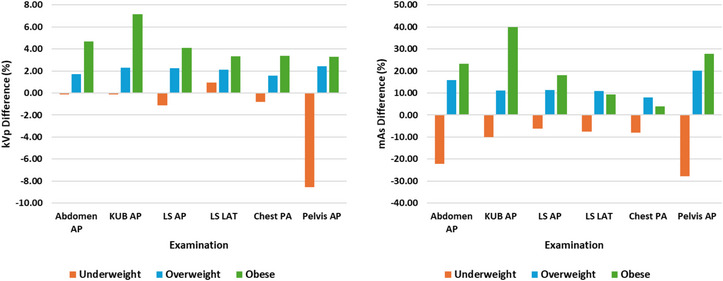
Percentage differences in kVp and mAs across BMI groups (underweight, overweight, and obese) for six projection radiography examinations. BMI, body mass index; kVp, kilovoltage peak; mAs, product of tube current and exposure time.

**FIGURE 2:**
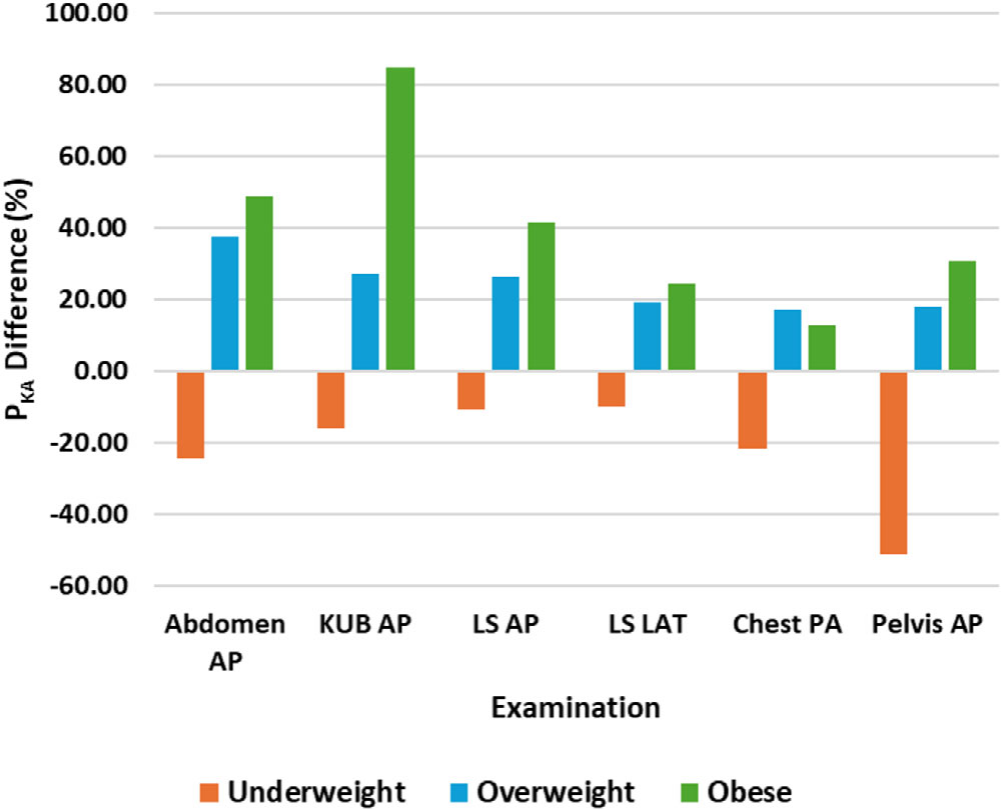
Percentage differences in P_KA_ across BMI groups (underweight, overweight, and obese) for six projection radiography examinations. BMI, body mass index; P_KA_, kerma‐area product.

Figure 1 and 2 show the percentage differences in kVp, mAs, and P_KA_ across BMI groups for six projection radiography examinations. The mean kVp values exhibited modest percentage increases for overweight (1.6%–2.4%) and obese (3.3%–7.2%) patients compared to normal weight patients for all examinations studied. Conversely, underweight patients showed percentage decreases in mean kVp (0.1%–8.6%) relative to normal weight patients. These findings suggest that kVp adjustments were relatively minor and primarily aimed at maintaining beam energy for consistent image quality across BMI categories.

In contrast, mAs values demonstrated more pronounced variations. Overweight patients exhibited percentage increases in mean mAs ranging from 8.0% to 20.2%, while obese patients displayed increases of 4.0% to 40.0%. Underweight patients showed decreases of 7.5% to 27.8% in mean mAs. These trends highlight that mAs adjustments are a primary factor in accommodating increased BMI while maintaining the appropriate diagnostic quality of radiographs.

The mean P_KA_ values revealed percentage increases for overweight patients, ranging from 17.4% to 37.9%, and for obese patients, ranging from 13.0% to 84.7%, when compared to normal weight patients across the examined studies. Conversely, underweight patients demonstrated percentage decreases in mean P_KA_, which varied from 10.0% to 51.3%, compared to normal weight patients in the same studies. These results emphasize that patient dose, as indicated by P_KA_, is substantially influenced by BMI, with higher doses required for overweight and obese patients to achieve adequate diagnostic quality for radiographs.

The progressive increase in kVp, mAs, and P_KA_ values as patients’ BMI increased from underweight to obese demonstrated that BMI significantly affects patient doses and exposure parameters, particularly mAs, which displayed the largest variations. For all examinations studied, underweight patients were subjected to considerably lower P_KA_ values compared to other BMI groups due to the use of lower mAs. Conversely, overweight and obese patients experienced significantly higher P_KA_ values than both underweight and normal weight patients due to increased mAs. This necessitates the use of higher exposure parameters with increased BMI, resulting in elevated P_KA_ values. These findings demonstrated that BMI is a critical determinant influencing dose variation in projection radiography examinations and were consistent with previous studies.^13^, ^20^, ^26^, ^27^

### 4.2 Comparison of exposure parameters: BMD approach versus DRL approach

This study underscores the need for BMI‐specific exposure parameters to address anatomical variability across different patient groups. Table 3 provides a comprehensive comparison of median exposure parameters for DRLs (58 ± 20 kg) and BMI‐based BMDs, revealing substantial differences, particularly in mAs values, across BMI groups. In this table, the median represents the 50th percentile value for each BMI group, while the median range indicates the variation in median exposure parameters utilized across individual hospitals. For example, in abdomen AP examinations, the median mAs ranged from 28 to 50 for underweight to obese patients in the BMI approach, whereas the median mAs varied from 32 to 40 across hospitals in the DRL approach. This indicates that using exposure parameters based on BMI intervals provides a more personalized approach for patient‐specific dose optimization.

**TABLE 3:** Comparison of median exposure parameters for DRLs and BMI‐based BMDs.

		kVp		mAs	
		BMD	DRL	BMD	DRL
Examination	BMI (kg m^−2^)	Median	Median range	Median	Median range
Abdomen AP (*N* = 86)	Underweight	70	66–74	28.0	32–40
	Normal weight	70		36.0	
	Overweight	72		45.0	
	Obese	75		50.0	
KUB AP (*N* = 198)	Underweight	70	68–76	32.0	32–50
	Normal weight	70		36.0	
	Overweight	70		40.0	
	Obese	75		53.0	
LS AP (*N* = 287)	Underweight	70	69–75	36.0	32–45
	Normal weight	70		36.0	
	Overweight	73		40.0	
	Obese	75		45.0	
LS LAT (*N* = 300)	Underweight	75	74–78	59.5	36–90
	Normal weight	75		63.0	
	Overweight	78		63.0	
	Obese	78		71.0	
Chest PA (*N* = 1060)	Underweight	100	95–110	5.0	2.5–5.6
	Normal weight	102		5.0	
	Overweight	104		5.0	
	Obese	105		5.0	
Pelvis AP (*N* = 58)	Underweight	70	67–75	20.0	28–40
	Normal weight	70		32.0	
	Overweight	70		36.0	
	Obese	75		41.0	

Abbreviations: AP, anteroposterior; BMD, benchmark dose; BMI, body mass index; DRL, diagnostic reference level; kVp, kilovoltage peak; LAT, lateral; LS, lumbar spine; mAs, product of tube current and exposure time; PA, posteroanterior.

Median kVp values showed minimal variation, with percentage increases of 0.0%–4.3% for overweight and 2.9%–7.1% for obese patients. This suggests that kVp adjustments were designed to preserve consistent beam energy across BMI groups, particularly for KUB AP and pelvis AP. Generally, BMI and body thickness exhibit a strong positive correlation, indicating that a higher BMI typically corresponds to greater body thickness, particularly in subcutaneous fat.^30^ This often requires adjusting the kVp for various BMI groups to penetrate the body effectively and achieve optimal image quality, which may lead to optimized patient doses.^30^ Conversely, mAs values exhibited more pronounced variations. Percentage increases in median mAs for overweight patients compared to normal weight patients were 25.0% for abdomen AP, 11.1% for KUB AP, 11.1% for lumbar spine AP, and 12.5% for pelvis AP. For obese patients, the increases were even higher, reaching 38.9%, 47.2%, 25.0%, 12.7%, and 28.1% for abdomen AP, KUB AP, lumbar spine AP, lumbar spine LAT, and pelvis AP, respectively. These trends highlight the primary role of mAs adjustments in compensating for increased attenuation associated with larger body sizes. Interestingly, chest PA examinations demonstrated no significant differences in median mAs among BMI groups. This finding indicates the need to optimize chest PA exposure parameters further, particularly for overweight and obese patients, without compromising the intended diagnostic information.

The observed trends reinforce the importance of using exposure parameters based on BMI intervals to support patient‐specific dose optimization. The kVp and mAs adjustments are essential for dose optimization in these examinations, which involve thicker anatomical regions in overweight and obese patients. For underweight patients, the application of lower kVp and mAs settings can further reduce radiation exposure without compromising diagnostic information. Conversely, higher kVp techniques, coupled with appropriately increased mAs values, should be implemented for overweight and obese patients to maintain image quality and patient doses within ALARP levels and minimize radiation‐induced stochastic risks.

These findings underscore the need for radiographers to replace preset exposure parameters (one size fits all patients) and instead adopt exposure parameters based on BMI intervals for patients with varying body habitus. In busy clinical settings, radiographers often default to pre‐set parameters planned for average‐sized patients, potentially leading to overexposure in smaller patients and underexposure in larger patients. This may degrade the intended diagnostic information in radiographs (image quality) and increase the risk of repeat examinations due to underexposure or overexposure (particularly if the detector becomes saturated). This highlights the importance of periodic protocol reviews to enhance awareness and promote the adoption of tailored exposure settings.

Moreover, factors beyond kVp and mAs, such as collimation and beam alignment, must be carefully optimized to account for variations in body habitus. Improved collimation techniques can significantly reduce scatter radiation and improve dose efficiency, particularly in obese patients. The AEC systems offer additional opportunities to streamline dose adjustments, although MEC modes require radiographers to make informed decisions based on patient‐specific parameters. It is expected that the calculated BMI‐based BMDs address anatomical variability more effectively than standard weight‐based parameters, ensuring personalized dose management and enhanced patient protection.

### 4.3 Formulation of BMI‐based BMDs

**FIGURE 3:**
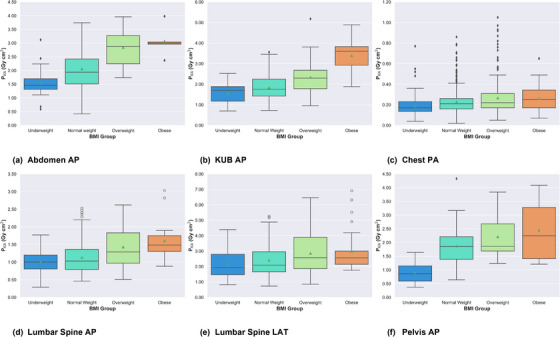
Boxplot representation of P_KA_ distribution among different BMI groups. BMI, body mass index; P_KA,_ kerma‐area product.

BMI‐based BMDs were formulated using median P_KA_ values for different BMI groups across all studied examinations. Figure 3 illustrates the distribution of P_KA_ values across BMI groups for all examinations studied. Median P_KA_ showed progressive increases with increasing BMI, indicating the impact of body habitus on patient doses. Compared to normal weight patients, underweight patients exhibited percentage reductions in BMI‐based BMDs of 24.7%, 3.4%, 2.9%, 7.1%, 4.5%, and 67.6% for abdomen AP, KUB AP, lumbar spine AP, lumbar spine LAT, chest PA, and pelvis AP, respectively. Conversely, overweight patients demonstrated percentage increases of 48.5%, 30.7%, 25.2%, 23.0%, 4.8%, and 0.5% across the same examinations, while the corresponding increases among obese patients were 54.6%, 104.5%, 51.5%, 22.5%, 19.0%, and 21.1%, respectively. These results underscore the necessity of BMI‐based BMDs as a more personalized approach for optimizing patient radiation exposure while maintaining appropriate diagnostic information.

### 4.4 Comparison of BMI‐based BMDs with DRLs

The comparison of BMI‐based BMDs with DRLs, as summarized in Table 4, highlights the importance of BMI‐specific dose optimization in projection radiography examinations of major anatomical regions. The results reveal that BMI‐based BMDs for overweight and obese patients were consistently higher than DRLs across all studied examinations, reflecting the increased radiation doses required to maintain diagnostic image quality in larger patients.

**TABLE 4:** Comparison of BMI‐based BMDs with standard weight‐based DRLs.

			DRLs (Gy.cm^2^)	
Examination	BMI (kg m^−2^)	BMI‐based BMDs (Gy.cm^2^)	IDRLs	ADs
Abdomen AP	Underweight	1.46	1.42–2.42	1.82
	Normal weight	1.94		
	Overweight	2.88		
	Obese	3.00		
KUB AP	Underweight	1.70	1.51–2.86	2.03
	Normal weight	1.76		
	Overweight	2.30		
	Obese	3.60		
LS AP	Underweight	1.00	0.83–1.65	1.27
	Normal weight	1.03		
	Overweight	1.29		
	Obese	1.48		
LS LAT	Underweight	1.94	1.76–4.10	2.21
	Normal weight	2.09		
	Overweight	2.57		
	Obese	2.56		
Chest PA	Underweight	0.21	0.10–0.26	0.22
	Normal weight	0.22		
	Overweight	0.25		
	Obese	0.60		
Pelvis AP	Underweight	0.60	1.32–2.74	1.90
	Normal weight	1.85		
	Overweight	1.86		
	Obese	2.24		

Abbreviations: ADs, achievable doses; AP, anteroposterior; BMD, benchmark dose; DRL, diagnostic reference level; BMI, body mass index; IDRLs, institutional DRLs; LAT, lateral; LS, lumbar spine; PA, posteroanterior.

BMI‐based BMDs for abdomen AP demonstrated progressive increases from 1.46 Gy.cm^2^ in underweight patients to 3.00 Gy.cm^2^ in obese patients. In comparison, the DRL for abdomen AP was 1.82 Gy.cm^2^, which aligns closely with the BMI‐based BMD for normal weight patients (1.94 Gy.cm^2^) but underestimates the dose delivered for overweight (2.88 Gy.cm^2^) and obese patients (3.00 Gy.cm^2^).

Similarly, KUB AP exhibited BMI‐based BMDs ranging from 1.70 to 3.60 Gy.cm^2^, whereas the DRL was 2.03 Gy.cm^2^. This corresponds to a 13.3% underestimation for overweight patients and a 43.7% underestimation for obese patients, underscoring the need for BMI‐specific dose optimization.

For lumbar spine AP, BMI‐based BMDs ranged from 1.00 Gy.cm^2^ in underweight patients to 1.48 Gy.cm^2^ in obese patients, compared to a DRL of 1.27 Gy.cm^2^. Lumbar spine LAT also demonstrated BMI‐based BMDs ranging from 1.94 to 2.56 Gy.cm^2^, compared to the DRL of 2.21 Gy.cm^2^. These findings indicate that while DRLs are reasonable for normal weight patients, they fail to account for the higher doses required for overweight and obese patients.

Chest PA demonstrated smaller variations, with BMI‐based BMDs ranging from 0.21 to 0.60 Gy.cm^2^, compared to a DRL of 0.22 Gy.cm^2^. Pelvis AP examinations showed BMI‐based BMDs ranging from 0.60 to 2.24 Gy.cm^2^, compared to a DRL of 1.90 Gy.cm^2^. The 17.9% increase observed in obese patients relative to the standard DRL highlights the need for additional dose optimization strategies in imaging regions with high anatomical attenuation.

These findings emphasize that BMI‐based BMDs offer a more appropriate and personalized framework for dose optimization compared to DRLs in terms of patient weight. Overweight and obese patients require significantly higher radiation doses to achieve acceptable image quality, which is inadequately accounted for in the protocols used for DRLs.^20^ Conversely, underweight patients may be exposed to unnecessarily high doses under standard protocols, reinforcing the need for tailored dose adjustments.

### 4.5 Practical implications of BMI‐based BMDs

This study highlights the pivotal role of BMI‐based BMDs in enhancing dose optimization for projection radiography examinations. Unlike DRLs, BMI‐based BMDs account for variations in patient body habitus and anatomical composition, providing a more personalized approach to dose optimization.^19^ This framework not only improves diagnostic accuracy but also improves patient protection by addressing the specific imaging needs of underweight, overweight, and obese patients in radiographic practice.

In clinical practice, accurately measuring anatomical thickness can be a practical challenge due to heavy workloads. In such cases, BMI can be used as an effective indicator of body size for selecting exposure parameters, as it has a strong positive correlation with body thickness, with a higher BMI typically corresponding to greater body thickness. This suggests that adjusting the kVp and mAs for different BMI groups to achieve optimal image quality may result in optimized patient doses, highlighting the important role of BMI‐based BMDs in routine practice.

BMI‐based BMDs mitigate the risks of underexposure in larger patients and overexposure in smaller patients, supporting sufficient image quality across BMI categories. For instance, higher P_KA_ values observed in obese patients necessitate protocol adjustments, including increased exposure parameters and optimized collimation, to compensate for greater attenuation.^15^ Conversely, underweight patients benefit from lower exposure settings, reducing unnecessary patient doses without compromising clinical information.

BMI‐based BMDs offer several practical benefits. First, they encourage radiographers to make informed decisions about exposure parameters, reducing the need to rely on pre‐set protocols designed for standard‐weight patients. Second, they facilitate the periodic evaluation of P_KA_ values, which supports continuous dose optimization for patients with varying body types. Third, regular monitoring enables the early identification of unusual radiographic practices, allowing for timely corrective actions and reviews.

To implement BMI‐based BMDs effectively, hospitals should prioritize targeted training programs for radiographers. These programs should emphasize the importance of tailoring exposure parameters to BMI groups and highlight techniques such as optimized collimation sizes for different BMI groups, post‐processing adjustments for larger patients, longer exposure times for obese patients, use of grid configurations, and accurate patient positioning to improve the diagnostic quality of radiographs.^31^, ^32^, ^33^

Technological advancements, including the development of automated software for calculating BMI‐based exposure parameters and phantom studies, can further streamline the exposure parameter selection process. Such tools can assist radiographers in selecting optimal kVp and mAs values and collimation settings based on BMI inputs, improving clinical workflow in hospitals with MEC settings. Future research should focus on evaluating image quality metrics alongside patient doses to establish a more comprehensive approach to dose optimization. Studies exploring the long‐term impact of BMI‐based dose management on patient outcomes will also provide valuable insights into its clinical effectiveness.

BMI‐based BMDs represent a significant advancement in radiographic imaging practices, accounting for anatomical variations in dose optimization. Even though BMI‐based BMDs address the limitations of DRLs, both approaches complement each other to improve dose optimization and patient protection. Therefore, the integration of BMI‐based BMDs alongside DRLs into clinical workflows enables safer and more effective radiographic practice, ultimately improving patient care outcomes.

## 5 LIMITATIONS

This study has a few limitations that need to be acknowledged due to practical constraints. The impact of field size on P_KA_ values, which could affect P_KA_ measurements, was not assessed. Even though BMI was calculated, the anatomical thickness of the imaged region was not measured. Differences in equipment performance and operator techniques across hospitals may have affected results. The relatively small sample sizes in the underweight and obese groups for certain examinations should be addressed. Furthermore, all radiographs were only qualitatively assessed by both radiographers and radiologists to ensure they were diagnostically acceptable for clinical purposes; however, no quantitative assessment was conducted.

## 6 CONCLUSIONS

This study underscores the importance of BMI‐based BMDs alongside DRLs as tools for dose optimization in projection radiography examinations. The findings highlight significant dose variations across BMI groups, necessitating tailored imaging protocols to accommodate differences in body habitus. By incorporating BMI‐specific benchmarks, radiographers can enhance patient protection and promote good radiographic practice. As the first of its kind in Sri Lanka, these findings hold significant potential to guide investigators and regulators attempting to establish DRLs in intervals of BMI in projection radiography examinations. The study methodology introduced here provides a robust guideline and template for other investigators worldwide. Future studies should focus on refining techniques and expanding datasets to further strengthen these recommendations. The integration of DRLs in intervals of BMI into clinical practice represents a pivotal step toward personalized radiation protection strategies, which face similar challenges in both Sri Lanka and other countries. Furthermore, the findings underscore the need for the introduction of international guidelines for DRLs in intervals of BMI to ensure standardized implementation across countries.

## AUTHOR CONTRIBUTIONS

**Sachith Welarathna**: Conceptualization; methodology, software; formal analysis; investigation; data curation; writing—original draft; writing—review & editing; visualization; project administration. **Sivakumar Velautham**: Conceptualization; methodology; writing—review & editing; resources; supervision; funding acquisition (APC). **Sivananthan Sarasanandarajah**: Conceptualization; methodology; writing—review & editing; resources; supervision. All authors read and approved the final manuscript.

## CONFLICT OF INTEREST STATEMENT

The authors declare no conflicts of interest.

## ETHICS STATEMENT

This study was performed in accordance with the Declaration of Helsinki. This human study was approved by the ethical review committee (ERC) of the National Hospital of Sri Lanka (AAJ/ETH/COM/2022). Each patient was informed about participation in the study, and verbal informed consent was obtained from all participants before data collection.

## Data Availability

The data that support the findings of this study are available from the corresponding author upon reasonable request.
